# Predictors of prolonged hospital stay in HIV-positive patients presenting to the emergency department

**DOI:** 10.1371/journal.pone.0249706

**Published:** 2021-04-21

**Authors:** Abdullah E. Laher, Fathima Paruk, Guy A. Richards, Willem D. F. Venter

**Affiliations:** 1 Faculty of Health Sciences, Department of Emergency Medicine, University of the Witwatersrand, Johannesburg, South Africa; 2 Department of Critical Care, University of Pretoria, Pretoria, South Africa; 3 Faculty of Health Sciences, Department of Critical Care, University of the Witwatersrand, Johannesburg, South Africa; 4 Faculty of Health Sciences, Ezintsha, University of the Witwatersrand, Johannesburg, South Africa; Rutland Regional Medical Center, UNITED STATES

## Abstract

**Background:**

Prolonged hospitalization places a significant burden on healthcare resources. Compared to the general population, hospital length of stay (LOS) is generally longer in HIV-positive patients. We identified predictors of prolonged hospital length of stay (LOS) in HIV-positive patients presenting to an emergency department (ED).

**Methods:**

In this cross-sectional study, HIV-positive patients presenting to the Charlotte Maxeke Johannesburg Academic Hospital adult ED were prospectively enrolled between 07 July 2017 and 18 October 2018. Data was subjected to univariate and multivariate logistic regression to determine parameters associated with a higher likelihood of prolonged hospital LOS, defined as ≥7 days.

**Results:**

Among the 1224 participants that were enrolled, the median (IQR) LOS was 4.6 (2.6–8.2) days, while the mean (SD) LOS was 6.9 (8.2) days. On multivariate analysis of the data, hemoglobin <11 g/dL (OR 1.37, p = 0.032), Glasgow coma scale (GCS) <15 (OR 1.80, p = 0.001), creatinine >120 μmol/L (OR 1.85, p = 0.000), cryptococcal meningitis (OR 2.45, p = 0.015) and bacterial meningitis (OR 4.83, p = 0.002) were significantly associated with a higher likelihood of LOS ≥7 days, while bacterial pneumonia (OR 0.35, p = 0.000) and acute gastroenteritis (OR 0.40, p = 0.025) were significantly associated with a lower likelihood of LOS ≥7 days.

**Conclusion:**

Various clinical and laboratory parameters are useful in predicting prolonged hospitalization among HIV-positive patients presenting to the ED. These parameters may be useful in guiding clinical decision making and directing the allocation of resources.

## Introduction

South Africa has an estimated 7.5 million people living with HIV (PLWH), which equates to approximately one-fifth of the global burden of the disease [1]. With the widespread availability of antiretroviral therapy (ART) over recent years, there has been a significant reduction in HIV-related hospitalization rates, hospital length of stay (LOS) and mortality [2,3]. Despite this, HIV still ranked as the 3^rd^ highest cause of global mortality in 2019 [1,4]. Furthermore, HIV contributes significantly to healthcare costs [5].

Compared to those attending HIV clinics, HIV-positive patients presenting to the emergency department (ED) tend to be more severely ill and frequently require hospitalization and admission to the intensive care unit (ICU) [6,7]. The average hospital LOS among HIV-positive patients requiring admission varies widely from approximately 4 days to over 15 days [7–10]. Compared to HIV-negative patients, those with HIV have higher rates of hospitalization and a longer LOS [8]. Besides being associated with poorer clinical outcomes, prolonged LOS also places a significant burden on healthcare resources. In fact, a large proportion of the overall financial burden relating to HIV has been attributed to the cost of in-hospital care and treatment of acutely ill HIV-positive patients [2,7].

There is a paucity of published data pertaining to factors that predict the length of hospitalization in HIV-positive patients presenting to the ED [11,12]. In addition to guiding clinical management, the identification of these factors may assist clinicians with triage and the appropriate channeling of resources, particularly in resource limited environments. Therefore, the aim of this study was to determine predictors of prolonged hospital stay (LOS ≥7 days) in HIV-positive patients presenting to the ED. Findings pertaining to other aspects of this study have been published separately [13–15].

## Methods

This was a cross-sectional study that was conducted at the adult medical-ED unit of the Charlotte Maxeke Johannesburg Academic Hospital (CMJAH). CMJAH is a 1088 bed tertiary-level academic hospital affiliated to the University of the Witwatersrand. The adult medical ED unit manages all non-trauma patients that are ≥16 years of age. On arrival to the ED triage area, patients are briefly assessed and categorized as either “emergent” (red), “very urgent” (orange), “urgent” (yellow) or “routine” (green), based on specific criteria as defined by the South African Triage Scale [16]. Since CMJAH is a tertiary level facility, in general patients that are categorized as red, orange, or yellow are managed at the facility, while stable patients that are categorized as green are referred to an alternate facility. Additionally, clinically stable patients not residing within the drainage area of the facility are also referred to an alternate facility closer to the patient’s residence.

As per the facility protocol, besides patients that are already HIV-positive (either self-reported or confirmed on laboratory records of patients that previously attended the facility), all other patients attending the ED are offered HIV-rapid diagnostic testing to determine their HIV status. Whole blood samples of patients consenting to HIV testing are tested with the Abon HIV 1/2/0 Tri-line Rapid test (Abon Biopharm, Hangzhou, RR China), with reactive samples thereafter subjected to a second confirmatory rapid test (First Response HIV 1–2.0 card, PMC Medical India Pvt, Ltd, Daman, India). In those in whom the first test is positive, but the confirmatory test is negative, a sample of whole blood is collected and sent to the laboratory for ELISA HIV-testing.

Data collection commenced once ethics clearance (University of the Witwatersrand Human Research Ethics Committee- clearance certificate number M160512) and relevant permissions were obtained. The ethics application included participant consent procedures that were also reviewed and approved by the committee. Adult patients ≥18 years that previously tested positive for HIV as well as those newly diagnosed with HIV after undergoing testing in the ED were prospectively enrolled into the study between 07 July 2017 and 18 October 2018. This included HIV-positive patients that required admission as well as patients that were directly discharged from the ED but excluded patients that were referred to another facility from the triage section. In addition, HIV-negative patients, HIV-status unknown patients who did not consent to HIV testing and patients not consenting to study participation were excluded.

Prior to the commencement of data collection, informal training pertaining to the methodology and principles of data collection from medical charts was undertaken by the primary investigator. After briefing all doctors employed in the ED regarding the study aim, objectives, and design they were requested to inform the primary investigator of all HIV-positive patients being managed in the ED. Written informed consent for study participation was obtained from potential participants by either the primary investigator or the doctor on shift. In the event that participants were unable to grant consent (e.g., decreased level of consciousness), consent was obtained from the next of kin/legal guardian and later re-obtained from the participant in the event that their mental capacity had improved. ED registers were also reviewed daily in an effort to identify potential participants that may have been missed by the ED doctors. For the purpose of this study, prolonged hospitalization was defined as LOS ≥7 days.

The four question AIDS Clinical Trials Group Adherence Questionnaire (ACTG-AQ) was utilized to determine non-adherence to ART [17]. Patients that responded “yes” to any of the questions were regarded as being ART non-adherent. The questionnaire was administered to all participants who had been prescribed ART at any time in the past.

Data was extracted from the patient’s hospital file by the primary investigator and electronically entered into an anonymized and standardized data collection form that was created in the RedCap system [18]. Additional information relevant to the study but not found in the patient’s hospital records was directly obtained from the participant, the participants laboratory records, or the participants next of kin/legal guardian where applicable. Only where the next of kin/legal guardian indicated that they were aware of the participant’s HIV status, they were questioned regarding relevant HIV-history such as treatment adherence. Data from hospital records were collected daily over the entire duration of hospital stay or until data collection was completed. Inter-rater reliability was assessed by an independent researcher experienced in the methods of data collection and blinded to the study aims and objectives. Data extracted from a random sample of 43 medical charts were compared to data extracted by the primary investigator.

Data relevant to this study included demographic details, HIV status, prior ART history including non-adherence, vital signs at presentation, results of relevant laboratory tests that were performed during the current presentation, presenting diagnoses, number of organ systems affected at presentation, disposition from the ED, length of hospital stay and in-hospital mortality. The vital signs data were also used to calculate the quick Sequential Organ Failure Assessment (qSOFA) score and the National Early Warning Score 2 (NEWS-2). The qSOFA score combines three rapid bedside clinical criteria (GCS <15, respiratory rate ≥22 breaths per minute and systolic blood pressure ≤100 mmHg) and is aimed at identifying patients that are at higher risk of in-hospital mortality [19]. The NEWS-2 score combines six criteria (respiratory rate, oxygen saturation, temperature, systolic blood pressure, heart rate and level of consciousness) and is aimed at determining severity of illness and prompting critical care interventions in patients being monitored in hospital [20]. The various presenting diagnoses were either microbiologically or histologically confirmed or were deemed as the most likely diagnosis based on findings of clinical assessment and special investigations and after discussion with the relevant sub-specialty clinician.

Data was exported to Microsoft^®^ Excel^®^ (Microsoft 365, Version 16.0.13029.20232) and thereafter to Stata version 16 (StataCorp Limited, Texas, United States of America) for statistical analysis. Besides age, all other continuous variables were categorized based on cut-offs frequently reported in the literature (e.g., CD_4_ cell count <100 cell/mm^3^; albumin <35 g/L etc.). Linearity was assessed using scatterplots. Frequency and percentage were determined for categorical variables. Depending on the number of participants in each group, either the Pearson’s chi-square or the Fisher’s exact test was used to determine if there were significant differences between the two groups.

The data was further subjected to univariate as well as multivariate analysis to determine factors influencing LOS ≥7 days while accounting for possible confounders such as age. On univariate analysis, binary logistic regression was used to determine factors associated with LOS ≥7 days. Crude odds ratio (OR) was reported with 95% confidence interval (CI) and p-value. For each of the variables assessed in the univariate analysis, all available case information was utilized. In the multivariate model, patients with missing data for the included variables were dropped from the model. All variables with a p-value of <0.1 in the univariate analysis were evaluated in the multivariate analysis. Non-significant variables were dropped with stepwise backward regression. To assess for interactions, interaction terms were individually added between variables in the multivariate model. Co-linearity was assessed via the Variance Inflation Factor (VIF) with values of >10 regarded as indicative of multicollinearity. A two-sided p-value of <0.05 was considered significant throughout. Study reporting was in conformance with STROBE (Strengthening the Reporting of Observational Studies in Epidemiology) guidelines [21].

## Results

A total of 11 383 of 29 416 patients that presented to the triage area of the adult medical ED over the period of data collection were managed in the ED, with the remainder being referred to an alternate appropriate facility as per the departmental triage protocol. Among those that were managed in the ED, 1308 were HIV-positive, however, 84 did not consent to study participation. Hence, the final study sample comprised 1224 patients [13–15]. The median (IQR) and mean (SD) LOS was 4.6 (2.6–8.2) days and 6.9 (8.2) days, respectively. The LOS was ≥7 days in 394 (32.2%) participants.

Comparisons and OR for LOS ≥7 days with respect to demographic details, HIV diagnosis, ART initiation/adherence, vital signs and laboratory parameters are described in Table 1. There were no statistically significant differences between participants with LOS ≥7 days and those with LOS <7 days (p <0.05) with regards to demographic characteristics and data pertaining to HIV diagnosis and ART initiation/adherence. With regards to vital signs at presentation, the likelihood of LOS ≥7 days was lower (OR 0.66, p = 0.021) among those presenting with an oxygen saturation <90%, while it was more than two times higher (OR 2.36, p <0.001) among those with a GCS <15. There were no statistically significant differences with regards to the other vital signs parameters that were analyzed. With regards to laboratory findings, the likelihood of LOS ≥7 days was higher among those presenting with a lactate >2.0 mmol/L (OR 1.32, p = 0.029), an albumin <35 g/L (OR 1.33, p = 0.039), a hemoglobin <11 g/dL (OR 1.50, p = 0.002), a urea >10 mmol/L (OR 1.97, p <0.001) and a creatinine >120 μmol/L (OR 1.98, p <0.001). There were no statistically significant differences with regards to any other laboratory parameters that were analyzed.

**Table 1:** Comparison of LOS with respect to demographic details, HIV diagnosis, ART initiation/adherence, vital signs, and laboratory parameters of study participants.

	Entire cohort	Length of admission		OR (95% CI)	P-value
		<7 days	≥7 days		
***Demographic characteristics***					
Age (years) [median (IQR)]	36 (31–44)	36 (30–43)	37 (31–44)	0.99 (0.98–1.01)	0.279
Sex [n (%)]					0.868
Female	673 (55.0)	447 (53.9)	226 (57.4)	1.00 (Reference)	
Male	551 (45.0)	383 (46.1)	168 (42.6)	0.87 (0.68–1.10)	
Race [n (%)]					0.119
Black	1174 (95.9)	791 (95.3)	383 (97.2)	1.00 (Reference)	
^a^ Other	50 (4.1)	39 (4.7)	11 (2.8)	0.58 (0.30–1.15)	
***HIV diagnosis and ART initiation/adherence***						
Newly diagnosed HIV [n (%)]	212 (17.3)	150 (18.1)	62 (15.7)	0.84 (0.61–1.17)	0.313
ART initiated prior to ED presentation [n (%)]	761 (62.2)	508 (61.2)	253 (64.2)	1.13 (0.89–1.46)	0.311
ART non-adherent [n (%)]	245 (32.2)	154 (30.3)	91 (23.1)	1.32 (0.98–1.77)	0.064
***Vital signs***					
Respiratory rate >20 breaths/min [n (%)]	434 (38.8)	303 (39.1)	131(38.1)	0.95 (0.74–1.24)	0.736
Oxygen saturation <90% [n (%)]	196 (17.5)	149 (19.3)	47 (13.6)	0.66 (0.46–0.94)	**0.021**
Systolic blood pressure <90 mmHg [n (%)]	116 (10.4)	81 (10.5)	35 (10.2)	0.97 (0.64–1.47)	0.878
Heart rate >110 beats/min [n (%)]	565 (50.6)	379 (49.1)	186 (53.9)	1.21 (0.94–1.56)	0.137
Glasgow coma scale <15 [n (%)]	221 (19.2)	117 (14.8)	104 (29.1)	2.36 (1.75–3.19)	**<0.001**
***Laboratory findings***					
CD_4_ <100 cell/mm^3^ [n (%)]	527 (47.6)	344 (46.0)	183 (51.1)	1.23 (0.95–1.58)	0.110
HIV viral load >1000 copies/mL [n (%)]	619 (59.0)	428 (59.4)	191 (58.2)	0.95 (0.73–1.24)	0.730
Hemoglobin <11 g/dL [n (%)]	579 (51.3)	374 (48.1)	205 (58.2)	1.50 (1.17–1.94)	**0.002**
White cell count <4.0 x 10^9^/L [n (%)]	170 (15.1)	117 (15.1)	53 (15.1)	1.01 (0.71–1.43)	0.971
Platelet count <150 x 10^9^/L [n (%)]	223 (19.9)	155 (20.1)	68 (19.5)	0.97 (0.70–1.33)	0.843
Urea >10 mmol/L [n (%)]	277 (25.9)	159 (21.7)	118 (35.2)	1.97 (1.48–2.61)	**<0.001**
Creatinine >120 μmol/L [n (%)]	300 (24.5)	173 (23.8)	127 (38.1)	1.98 (1.50–2.62)	**<0.001**
C-reactive protein >100 mg/L [n (%)]	516 (48.7)	339 (47.0)	177 (52.5)	1.25 (0.96–1.62)	0.092
Lactate >2 mmol/L [n (%)]	470 (42.0)	307 (39.9)	163 (46.8)	1.32 (1.03–1.71)	**0.029**
Albumin <35 g/L [n (%)]	633 (60.7)	418 (58.6)	215 (65.3)	1.33 (1.01–1.75)	**0.039**
Alanine transaminase >100 mmol/L [n (%)]	109 (10.6)	70 (10.0)	39 (11.9)	1.22 (0.80–1.84)	0.356

OR–Odds ratio, ART–antiretroviral therapy, ED–emergency department.

Bold–denotes statistical significance.

^a^ Includes Asian, Caucasian, and mixed race.

Comparisons and OR for LOS ≥7 days with respect to common HIV-related presenting diagnoses, illness severity scores, ICU admission and in-hospital mortality are described in Table 2. With regards to the presenting diagnoses, the likelihood of LOS ≥7 days was higher in those that presented with tuberculosis (OR 1.66, p, 0.001), with participants that were diagnosed with abdominal tuberculosis (OR 2.31, p = 0.031) and tuberculous meningitis (OR 4.07, p = 0.002) showing the highest likelihood of LOA ≥7 days. Among those with cryptococcal and bacterial meningitis, the likelihood for LOS ≥7 days was approximately two and a half times (OR 2.54, p = 0.004) and just over six times (OR 6.08, p <0.001) higher, respectively. The likelihood for LOS ≥7 days was lower among those with bacterial pneumonia (OR 0.37, p <0.001).

**Table 2:** Comparison of LOS with respect to common HIV related presenting diagnoses, illness severity scores, ICU admission and in-hospital mortality of study participants.

	Entire cohort (n, %)	Length of admission		OR (95% CI)	P-value
		<7 days (n, %)	≥7 days (n, %)		
Tuberculosis	244 (19.9)	143 (17.2)	101 (25.6)	1.66 (1.24–2.21)	**<0.001**
Single organ tuberculosis	174 (14.2)	103 (12.4)	71 (18.0)	1.62 (1.16–2.25)	**0.005**
Disseminated tuberculosis	70 (5.7)	40 (4.8)	30 (7.6)	1.63 (1.00–2.66)	**0.049**
^a^ Pulmonary tuberculosis	101 (8.3)	65 (7.8)	36 (9.1)	1.18 (0.77–1.81)	0.438
Extrapulmonary tuberculosis	143 (11.7)	78 (9.4)	65 (16.5)	1.90 (1.34–2.71)	**<0.001**
Miliary tuberculosis	38 (3.1)	21 (2.5)	17 (4.3)	1.74 (0.91–3.33)	0.096
Pleural tuberculosis	31 (2.5)	23 (2.8)	8 (2.0)	0.73 (0.32–1.64)	0.443
Abdominal tuberculosis	27 (2.2)	13 (1.6)	14 (3.6)	2.31 (1.08–4.97)	**0.031**
Tuberculous meningitis	23 (1.9)	8 (1.0)	15 (3.8)	4.07 (1.71–9.67)	**0.002**
^**b**^ Other	29 (2.4)	16 (1.9)	13 (3.3)	1.74 (0.83–3.65)	0.145
Bacterial pneumonia	276 (22.5)	228 (27.5)	48 (12.2)	0.37 (0.26–0.51)	**<0.001**
Pneumocystis jirovecii pneumonia	47 (3.8)	37 (4.4)	10 (2.5)	0.56 (.027–1.13)	0.11
Cryptococcal meningitis	39 (3.2)	18 (2.2)	21 (5.3)	2.54 (1.34–4.82)	**0.004**
Bacterial meningitis	30 (2.5)	8 (1.0)	22 (5.6)	6.08 (2.68–13.78)	**<0.001**
Acute gastroenteritis	56 (4.6)	44 (5.3)	12 (3.0)	0.56 (0.29–1.07)	0.08
Chronic gastroenteritis	30 (2.5)	19 (2.2)	11 (2.8)	1.23 (0.58–2.60)	0.60
No. of organ systems affected					
1	460 (37.6)	324 (39.0)	136 (34.5)	1.00 (Reference)	
2	432 (35.2)	302 (36.4)	130 (33.0)	1.03 (0.77–1.37)	0.863
≥3	332 (27.2)	204 (24.6)	128 (32.5)	1.49 (1.11–1.01	**0.008**
qSOFA score					
Low score (0–1 point)	921 (82.5)	642 (82.9)	279 (81.3)	1.00 (Reference)	
High score (2–3 points)	196 (17.5)	132 (17.2)	64 (18.7)	1.12 (0.80–1.55)	0.516
NEWS-2 score					
Low score (0–4 points)	449 (40.2)	316 (40.9)	133 (38.7)	1.00 (Reference)	
^c^ Medium score (5–6 points)	171 (15.4)	111 (14.4)	60 (17.4)	1.28 (0.88–1.87)	0.189
High score (≥7 points)	496 (44.4)	345 (44.7)	151 (43.9)	1.04(0.79–1.37)	0.783
ICU admission	205 (16.7)	117 (14.1)	88 (22.3)	1.75 (1.29–2.38)	**<0.001**
In-hospital mortality	166 (13.6)	98 (11.8)	68 (17.2)	1.56 (1.11–2.18)	**0.010**

OR–Odds ratio.

Bold–denotes statistical significance.

^a^ Only includes participants with isolated pulmonary tuberculosis.

^**b**^ Tuberculous lymphadenitis (n = 10), tuberculous pericarditis (n = 9), tuberculoma (n = 4), urogenital tuberculosis (n = 3), spinal tuberculosis (n = 2), tuberculous osteomyelitis (n = 1).

^**c**^ Includes participants with an overall low score but achieving 3 points in any individual parameter.

Compared to participants presenting with pathology affecting a single organ system, there was no significant difference in LOS ≥7 days among those presenting with pathology affecting two organ systems (p = 0.863), but however, those with pathology affecting ≥3 organ systems had almost a one and half times higher likelihood of LOS ≥7 days (OR 1.49, p = 0.008).

The qSOFA and NEWS-2 scores were not significantly associated with LOS ≥7 days (p >0.05 for both), however, the likelihood of LOS ≥7 days was higher among those that required ICU admission (OR 1.75, p <0.001) as well as among those that died during hospital admission (OR 1.56, p = 0.010).

After adjusting for oxygen saturation and removing participants with missing data, variables with a p-value <0.1 from the univariate analysis were subjected to multivariate logistic regression. A total of 1027 (83.9%) participants were included in the final model (Table 3). Hemoglobin <11 g/dL (OR 1.37, p = 0.032), GCS <15 (OR 1.80, p = 0.001), creatinine >120 μmol/L (OR 1.85, p = 0.000), cryptococcal meningitis (OR 2.45, p = 0.015) and bacterial meningitis (OR 4.83, p = 0.002) were significantly associated with a higher likelihood of LOS ≥7 days, while bacterial pneumonia (OR 0.35, p = 0.000) and acute gastroenteritis (OR 0.40, p = 0.025), were significantly associated with a lower likelihood of LOS ≥7 days.

**Table 3:** Variables significantly associated with LOS ≥7 days after subjecting the relevant data to multivariate analysis.

Parameter	Multivariate analysis	
	OR (95% CI)	P-value
Hemoglobin <11 g/dL	1.37 (1.03–1.83)	**0.032**
Glasgow coma scale <15	1.80 (1.28–2.54)	**0.001**
Creatinine >120 μmol/L	1.85 (1.36–2.52)	**0.000**
Cryptococcal meningitis	2.45 (1.19–5.05)	**0.015**
Bacterial meningitis	4.83 (1.82–12.82)	**0.002**
Bacterial pneumonia	0.35 (0.23–0.52)	**0.000**
Acute gastroenteritis	0.40 (0.18–0.89)	**0.025**

OR–Odds ratio.

Model adjusted for oxygen saturation.

## Discussion

To our knowledge, this is the largest and most comprehensive single-center study to have determined predictors of prolonged hospital admission in consecutive HIV-positive patients admitted via the ED. In fact, overall, there are very few studies that have reported parameters that predict prolonged hospital stay among HIV-positive patients and these studies have examined a very limited number of parameters. As such comparison of the findings of this study to others is difficult.

In 2 studies conducted in Rhode Island, USA [7] and Calgary, Canada [8] the median LOS was 4.7 and 5 days respectively among hospitalized HIV-positive patients, which was comparable with the median LOS in this study at 4.6 days. In contrast, in a longitudinal study of hospitalized HIV-positive patients conducted in Rio de Janeiro, Brazil, the authors reported a much longer median LOS of 15 days in 2007 and 11 days in 2013, with a longer median LOS in patients admitted with AIDS related illnesses than those admitted for non-AIDS related illnesses [3]. Although we did not directly compare patients with and without AIDS defining illnesses, subsets of patients with tuberculosis, cryptococcal meningitis and bacterial meningitis had a significantly higher likelihood of a prolonged LOS. Studies conducted in Portugal and Columbia among hospitalized HIV-positive patients also reported a longer median LOS of 12 [9] and 14 days [10] respectively.

With regard to the specific factors associated with a longer LOS, a longitudinal study conducted in Portugal between 2009–2014 found that the number of secondary diagnoses, the number of inpatient procedures, and tuberculosis were associated with a longer LOS, whereas female gender, emergent admission, in-hospital mortality, Pneumocystis jirovecii pneumonia and hepatitis C were all associated with a shorter LOS [22]. Furthermore, the study conducted in Calgary, Canada reported that a low CD_4_ cell count, presence of an AIDS defining illness and no current use of ART were strongly correlated with hospitalization (p <0.01) [8]. Additionally, a study that retrospectively evaluated the effect of various factors on hospital LOS in Louisiana, USA between 1998 and 2003, reported that gender and age were non-significant predictors, while the number of comorbid conditions and inpatient procedures and the presence of an AIDS defining diagnosis were significant independent predictors [11]. Another study conducted in Brazil, reported that low income was associated with prolonged LOS among HIV-positive patients with tuberculosis [12]. In comparison to the findings of some of the above studies, tuberculosis, multiple organ involvement (≥3 organ systems) and in-hospital mortality were associated with a longer LOS on univariate analysis, while there were no significant differences with regards to age, gender, low CD_4_ cell count (<100 cell/mm^3^), use of ART and Pneumocystis jirovecii pneumonia.

Although there is a lack of comparable data pertaining to HIV-positive patients and LOS, low GCS [23], anemia [24–26], elevated urea [27,28], elevated creatinine [28,29], hypalbuminemia [26,30], hyperlactatemia [31], cryptococcal meningitis [32] and bacterial meningitis [33] have been associated with prolonged LOS in multiple non-HIV based studies that enrolled general medical, perioperative and other disease-specific population groups.

It is rather surprising that those with oxygen saturation <90% had a significantly lower likelihood of prolonged LOS (OR 0.37, p <0.001). Although the underlying reasons for this finding is unclear and were not explored in this study, the large number of study participants that presented with bacterial pneumonia and other respiratory system pathologies (most of whom had a LOS <7 days), may be a possible explanation. Furthermore, it is also possible that some patients with a low oxygen saturation at presentation may have demised during the initial days of their hospital admission.

There are some limitations to this study. Firstly, this was a single center study, hence our findings may not necessarily be generalizable to other settings where criteria for patient discharge may differ. Secondly, it is possible that some patients may have been readmitted for the same illness shortly after having been discharged, which was not taken into account when calculating the LOS. Thirdly, some patients were discharged for continuation of care to a rehabilitation or step-down facility, which was also not accounted for when calculating the LOS. Finally, we did not account for the presence of underlying comorbid and other chronic illnesses, nor did we account for individual social circumstances, both of which may have also influenced the overall LOS.

## Conclusion

There are specific criteria that predict longer length of stay in HIV-positive patients presenting to the ED. These findings may be useful in guiding clinical decision making, directing the appropriate channeling of resources, and facilitating the design of innovative strategies to optimize LOS.

## Supporting information

S1 Dataset (XLSX)
